# Diaqua­bis­[4-(dimethyl­amino)­benzoato-κ*O*]bis­(nicotinamide-κ*N* ^1^)zinc(II) dihydrate

**DOI:** 10.1107/S1600536810046854

**Published:** 2010-11-24

**Authors:** Tuncer Hökelek, Ertuğrul Gazi Sağlam, Barış Tercan, Özgür Aybirdi, Hacali Necefoğlu

**Affiliations:** a Department of Physics, Hacettepe University, 06800 Beytepe, Ankara, Turkey; b Department of Chemistry, Ankara University, 06100 Tandoğan, Ankara, Turkey; c Department of Physics, Karabük University, 78050 Karabük, Turkey; d Department of Chemistry, Kafkas University, 36100 Kars, Turkey

## Abstract

In the centrosymmetric title structure, [Zn(C_9_H_10_NO_2_)_2_(C_6_H_6_N_2_O)_2_(H_2_O)_2_]·2H_2_O, the Zn^II^ cation, located on an inversion center, is coordinated by two 4-(methyl­amino)­benzoate anions, two nicotinamide ligands and two water mol­ecules in a slightly distorted octa­hedral geometry. The dihedral angle between the carboxyl­ate group and the attached benzene ring is 3.09 (9)°, while the pyridine and benzene rings are oriented at a dihedral angle of 77.10 (4)°. The uncoordinated water mol­ecule is linked to nicotinamide ligands by O—H⋯O hydrogen bonds. In the crystal, inter­molecular N—H⋯O, O—H⋯O and C—H⋯O hydrogen bonds link the mol­ecules into a three-dimensional network. A weak N—H⋯π inter­action also occurs.

## Related literature

For niacin, see: Krishnamachari (1974 ▶). For *N*,*N*-diethyl­nicotinamide, see: Bigoli *et al.* (1972 ▶). For related structures, see: Hökelek *et al.* (1996 ▶, 2009*a* ▶,*b* ▶,*c* ▶); Hökelek & Necefoğlu (1998 ▶); Necefoğlu *et al.* (2010*a* ▶,*b* ▶).
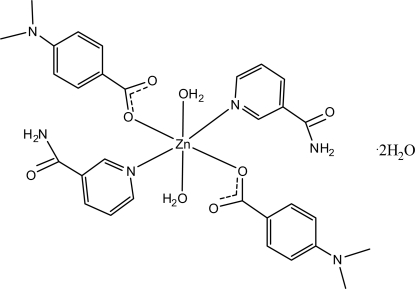


## Experimental

### 

#### Crystal data

[Zn(C_9_H_10_NO_2_)_2_(C_6_H_6_N_2_O)_2_(H_2_O)_2_]·2H_2_O
*M* *_r_* = 710.07
Triclinic, 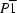

*a* = 8.1810 (2) Å
*b* = 9.9877 (2) Å
*c* = 10.1982 (3) Å
α = 76.141 (2)°
β = 88.894 (3)°
γ = 78.200 (2)°
*V* = 791.55 (4) Å^3^
*Z* = 1
Mo *K*α radiation
μ = 0.84 mm^−1^
*T* = 100 K
0.40 × 0.24 × 0.18 mm

#### Data collection

Bruker Kappa APEXII CCD area-detector diffractometer
Absorption correction: multi-scan (*SADABS*; Bruker, 2005 ▶) *T*_min_ = 0.783, *T*_max_ = 0.856
14586 measured reflections
3975 independent reflections
3725 reflections with *I* > 2σ(*I*)
*R*_int_ = 0.020

#### Refinement

*R*[*F* ^2^ > 2σ(*F* ^2^)] = 0.026
*wR*(*F* ^2^) = 0.068
*S* = 1.05
3975 reflections
240 parameters
H atoms treated by a mixture of independent and constrained refinement
Δρ_max_ = 0.32 e Å^−3^
Δρ_min_ = −0.21 e Å^−3^

### 

Data collection: *APEX2* (Bruker, 2007 ▶); cell refinement: *SAINT* (Bruker, 2007 ▶); data reduction: *SAINT*; program(s) used to solve structure: *SHELXS97* (Sheldrick, 2008 ▶); program(s) used to refine structure: *SHELXL97* (Sheldrick, 2008 ▶); molecular graphics: *ORTEP-3 for Windows* (Farrugia, 1997 ▶); software used to prepare material for publication: *WinGX* publication routines (Farrugia, 1999 ▶) and *PLATON* (Spek, 2009 ▶).

## Supplementary Material

Crystal structure: contains datablocks I, global. DOI: 10.1107/S1600536810046854/xu5088sup1.cif

Structure factors: contains datablocks I. DOI: 10.1107/S1600536810046854/xu5088Isup2.hkl

Additional supplementary materials:  crystallographic information; 3D view; checkCIF report

## Figures and Tables

**Table 1:** Selected bond lengths (Å)

Zn1—O1	2.0442 (9)
Zn1—O4	2.1503 (11)
Zn1—N1	2.1963 (10)

**Table 2:** Hydrogen-bond geometry (Å, °) *Cg*1 is the centroid of the C2–C7 ring.

*D*—H⋯*A*	*D*—H	H⋯*A*	*D*⋯*A*	*D*—H⋯*A*
N2—H21⋯O5^i^	0.85 (2)	2.05 (2)	2.8826 (19)	169.3 (2)
O4—H41⋯O2^ii^	0.78 (2)	2.00 (2)	2.7370 (16)	159 (2)
O4—H42⋯O3^iii^	0.81 (2)	1.96 (2)	2.7681 (15)	175.1 (2)
O5—H51⋯O2	0.85 (3)	2.02 (3)	2.8732 (19)	174 (3)
O5—H52⋯O2^iv^	0.81 (3)	2.11 (3)	2.9150 (18)	173 (2)
C13—H13⋯O4^v^	0.93	2.52	3.4422 (19)	170
N2—H22⋯*Cg*1^ii^	0.829 (19)	2.79 (2)	3.5200 (15)	147.9 (2)

Symmetry codes: (i) 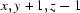; (ii) 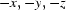; (iii) 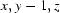; (iv) 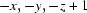; (v) 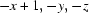.

**Fig. 1.**
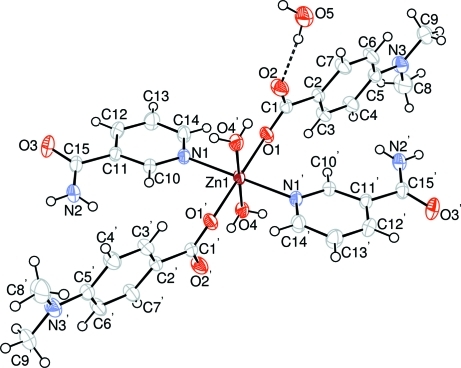
The molecular structure of the title compound with the atom-numbering scheme. Displacement ellipsoids are drawn at the 50% probability level. Primed atoms are generated by the symmetry operator: (') -x, -y, -z. Only one of the crystal water molecules is shown [dashed line indicates the O—H···O hydrogen-bond].

## supplementary crystallographic information

### Comment

As a part of our ongoing investigation on transition metal complexes of nicotinamide (NA), one form of niacin (Krishnamachari, 1974), and/or the nicotinic acid derivative *N*,*N*-diethylnicotinamide (DENA), an important respiratory stimulant (Bigoli *et al.*, 1972), the title compound was synthesized and its crystal structure is reported herein.

The title compound, (I), is a mononuclear complex, where the Zn^II^ ion is located on a crystallographic inversion center. The asymmetric unit contains one 4-(methylamino)benzoate (PMAB) anion, one nicotinamide (NA) ligand, one coordinated and one uncoordinated water molecules, all ligands are monodentate (Fig. 1). The crystal structures of some NA and/or DENA complexes of Cu^II^, Co^II^, Ni^II^, Mn^II^ and Zn^II^ ions, [Cu(C_7_H_5_O_2_)_2_(C_10_H_14_N_2_O)_2_], (II) (Hökelek *et al.*, 1996), [Cu_2_(C_8_H_7_O_2_)_4_(C_6_H_6_N_2_O)_2_], (III) (Necefoğlu *et al.*, 2010*a*), [Co(C_6_H_6_N_2_O)_2_(C_7_H_4_NO_4_)_2_(H_2_O)_2_], (IV) (Hökelek & Necefoğlu, 1998), [Ni(C_7_H_4_ClO_2_)_2_(C_6_H_6_N_2_O)_2_(H_2_O)_2_], (V) (Hökelek *et al.*, 2009*a*), [Ni(C_8_H_7_O_2_)_2_(C_6_H_6_N_2_O)_2_(H_2_O)_2_], (VI) (Necefoğlu *et al.*, 2010*b*), [Mn(C_7_H_4_ClO_2_)_2_(C_10_H_14_N_2_O)_2_(H_2_O)_2_], (VII) (Hökelek *et al.*, 2009*b*) and [Zn(C_7_H_4_BrO_2_)_2_(C_6_H_6_N_2_O)_2_(H_2_O)_2_], (VIII) (Hökelek *et al.*, 2009*c*) have also been reported. In (II), two benzoate ions are coordinated to the Cu atom as bidentate ligands, while in other structures all ligands being monodentate.

The four O atoms (O1, O4, and the symmetry-related atoms, O1', O4') in the equatorial plane around the Zn^II^ ion form a slightly distorted square-planar arrangement, while the slightly distorted octahedral coordination is completed by the two N atoms of the NA ligands (N1, N1') in the axial positions (Fig. 1). The near equality of the C1—O1 [1.2691 (16) Å] and C1—O2 [1.2624 (17) Å] bonds in the carboxylate group indicates a delocalized bonding arrangement, rather than localized single and double bonds. The average Zn—O bond length is 2.0973 (10) Å (Table 1), and the Zn^II^ ion is displaced out of the least-squares plane of the carboxylate group (O1/C1/O2) by -0.8557 (1) Å. The dihedral angle between the planar carboxylate group and the benzene ring A (C2—C7) is 3.09 (9)°, while that between rings A and B (N1/C10—C14) is 77.10 (4)°. The uncoordinated water molecules are linked to the NA ligands by O—H···O hydrogen bonds (Table 2 and Fig. 1).

In the crystal structure, intermolecular N—H···O, O—H···O, and C—H···O hydrogen bonds (Table 2) link the molecules into a three-dimensional network, in which they may be effective in the stabilization of the structure. There also exists a weak N-H···π interaction (Table 2).

### Experimental

The title compound was prepared by the reaction of ZnSO_4_.H_2_O (0.90 g, 5 mmol) in H_2_O (50 ml) and nicotinamide (1.22 g, 10 mmol) in H_2_O (30 ml) with sodium 4-dimethylaminobenzoate (1.88 g, 10 mmol) in H_2_O (100 ml). The mixture was filtered and set aside to crystallize at ambient temperature for one week, giving colorless single crystals.

### Refinement

Atoms H21, H22 (for NH_2_) and H41, H42, H51, H52 (for H_2_O) were located in a difference Fourier map and refined isotropically. The remaining H atoms were positioned geometrically with C—H = 0.93 and 0.96 Å for aromatic and methyl H atoms and constrained to ride on their parent atoms, with *U*_iso_(H) = *xU*_eq_(C), where *x* = 1.5 for methyl H and *x* = 1.2 for aromatic H atoms.

### Crystal data

**Table:**

[Zn(C_9_H_10_NO_2_)_2_(C_6_H_6_N_2_O)_2_(H_2_O)_2_]·2H_2_O	*Z* = 1
*M**_r_* = 710.07	*F*(000) = 372
Triclinic, *P*1	*D*_x_ = 1.490 Mg m^−^^3^
Hall symbol: -P 1	Mo *K*α radiation, λ = 0.71073 Å
*a* = 8.1810 (2) Å	Cell parameters from 7854 reflections
*b* = 9.9877 (2) Å	θ = 2.6–28.4°
*c* = 10.1982 (3) Å	µ = 0.84 mm^−^^1^
α = 76.141 (2)°	*T* = 100 K
β = 88.894 (3)°	Block, colorless
γ = 78.200 (2)°	0.40 × 0.24 × 0.18 mm
*V* = 791.55 (4) Å^3^	

### Data collection

**Table:**

Bruker Kappa APEXII CCD area-detector diffractometer	3975 independent reflections
Radiation source: fine-focus sealed tube	3725 reflections with *I* > 2σ(*I*)
graphite	*R*_int_ = 0.020
φ and ω scans	θ_max_ = 28.5°, θ_min_ = 2.1°
Absorption correction: multi-scan (*SADABS*; Bruker, 2005)	*h* = −10→10
*T*_min_ = 0.783, *T*_max_ = 0.856	*k* = −13→13
14586 measured reflections	*l* = −13→13

### Refinement

**Table:**

Refinement on *F*^2^	Primary atom site location: structure-invariant direct methods
Least-squares matrix: full	Secondary atom site location: difference Fourier map
*R*[*F*^2^ > 2σ(*F*^2^)] = 0.026	Hydrogen site location: inferred from neighbouring sites
*wR*(*F*^2^) = 0.068	H atoms treated by a mixture of independent and constrained refinement
*S* = 1.05	*w* = 1/[σ^2^(*F*_o_^2^) + (0.0322*P*)^2^ + 0.2491*P*] where *P* = (*F*_o_^2^ + 2*F*_c_^2^)/3
3975 reflections	(Δ/σ)_max_ < 0.001
240 parameters	Δρ_max_ = 0.32 e Å^−^^3^
0 restraints	Δρ_min_ = −0.21 e Å^−^^3^

### Special details

**Table:**

Geometry. All esds (except the esd in the dihedral angle between two l.s. planes) are estimated using the full covariance matrix. The cell esds are taken into account individually in the estimation of esds in distances, angles and torsion angles; correlations between esds in cell parameters are only used when they are defined by crystal symmetry. An approximate (isotropic) treatment of cell esds is used for estimating esds involving l.s. planes.
Refinement. Refinement of F^2^ against ALL reflections. The weighted R-factor wR and goodness of fit S are based on F^2^, conventional R-factors R are based on F, with F set to zero for negative F^2^. The threshold expression of F^2^ > 2sigma(F^2^) is used only for calculating R-factors(gt) etc. and is not relevant to the choice of reflections for refinement. R-factors based on F^2^ are statistically about twice as large as those based on F, and R- factors based on ALL data will be even larger.

### Fractional atomic coordinates and isotropic or equivalent isotropic displacement parameters (Å^2^)

**Table:**

	*x*	*y*	*z*	*U*_iso_*/*U*_eq_	
Zn1	0.0000	0.0000	0.0000	0.02409 (7)	
O1	0.14766 (12)	−0.13955 (9)	0.15299 (10)	0.0301 (2)	
O2	0.01781 (15)	−0.12085 (10)	0.34423 (11)	0.0406 (3)	
O3	0.22843 (14)	0.63006 (10)	−0.11818 (13)	0.0428 (3)	
O4	0.16532 (13)	−0.08200 (11)	−0.14162 (11)	0.0305 (2)	
H41	0.128 (3)	−0.035 (2)	−0.211 (2)	0.057 (6)*	
H42	0.179 (3)	−0.165 (2)	−0.138 (2)	0.052 (6)*	
O5	−0.1009 (2)	−0.13619 (14)	0.61334 (15)	0.0543 (3)	
H51	−0.070 (3)	−0.136 (3)	0.533 (3)	0.083 (9)*	
H52	−0.086 (3)	−0.063 (3)	0.628 (2)	0.069 (7)*	
N1	0.15651 (14)	0.15584 (11)	−0.00723 (11)	0.0266 (2)	
N2	0.00655 (17)	0.58552 (13)	−0.21424 (13)	0.0355 (3)	
H21	−0.027 (2)	0.671 (2)	−0.2554 (18)	0.040 (5)*	
H22	−0.047 (2)	0.529 (2)	−0.2306 (19)	0.041 (5)*	
N3	0.45941 (17)	−0.75023 (13)	0.50923 (14)	0.0395 (3)	
C1	0.11626 (17)	−0.18927 (13)	0.27526 (13)	0.0272 (3)	
C2	0.20133 (16)	−0.33741 (13)	0.33617 (13)	0.0261 (3)	
C3	0.31531 (17)	−0.41206 (14)	0.26313 (14)	0.0314 (3)	
H3	0.3353	−0.3688	0.1746	0.038*	
C4	0.39964 (18)	−0.54815 (15)	0.31784 (15)	0.0341 (3)	
H4	0.4749	−0.5949	0.2659	0.041*	
C5	0.37268 (17)	−0.61713 (13)	0.45193 (14)	0.0300 (3)	
C6	0.2540 (2)	−0.54300 (15)	0.52383 (15)	0.0373 (3)	
H6	0.2304	−0.5866	0.6114	0.045*	
C7	0.1715 (2)	−0.40668 (15)	0.46724 (14)	0.0347 (3)	
H7	0.0943	−0.3600	0.5178	0.042*	
C8	0.5794 (3)	−0.82465 (18)	0.4327 (2)	0.0539 (5)	
H8A	0.6301	−0.9151	0.4886	0.081*	
H8B	0.6640	−0.7712	0.4028	0.081*	
H8C	0.5241	−0.8373	0.3557	0.081*	
C9	0.4290 (2)	−0.82016 (16)	0.64594 (18)	0.0481 (4)	
H9A	0.5045	−0.9100	0.6712	0.072*	
H9B	0.3160	−0.8336	0.6515	0.072*	
H9C	0.4464	−0.7633	0.7061	0.072*	
C10	0.10521 (16)	0.29304 (13)	−0.06636 (13)	0.0254 (2)	
H10	−0.0026	0.3238	−0.1040	0.030*	
C11	0.20513 (16)	0.39157 (13)	−0.07418 (13)	0.0255 (2)	
C12	0.36499 (18)	0.34448 (15)	−0.01643 (16)	0.0333 (3)	
H12	0.4347	0.4076	−0.0181	0.040*	
C13	0.41990 (18)	0.20295 (16)	0.04360 (17)	0.0384 (3)	
H13	0.5273	0.1692	0.0816	0.046*	
C14	0.31197 (18)	0.11262 (14)	0.04593 (15)	0.0335 (3)	
H14	0.3491	0.0174	0.0863	0.040*	
C15	0.14646 (17)	0.54593 (13)	−0.13868 (14)	0.0290 (3)	

### Atomic displacement parameters (Å^2^)

**Table:**

	*U*^11^	*U*^22^	*U*^33^	*U*^12^	*U*^13^	*U*^23^
Zn1	0.02776 (11)	0.01681 (10)	0.02584 (11)	−0.00591 (7)	−0.00232 (8)	−0.00020 (7)
O1	0.0319 (5)	0.0245 (4)	0.0288 (5)	−0.0056 (4)	−0.0030 (4)	0.0035 (4)
O2	0.0583 (7)	0.0255 (5)	0.0311 (5)	0.0038 (4)	0.0011 (5)	−0.0040 (4)
O3	0.0468 (6)	0.0225 (5)	0.0621 (7)	−0.0132 (4)	0.0022 (5)	−0.0111 (5)
O4	0.0348 (5)	0.0206 (4)	0.0342 (6)	−0.0030 (4)	−0.0019 (4)	−0.0046 (4)
O5	0.0850 (10)	0.0309 (6)	0.0437 (8)	−0.0101 (6)	−0.0006 (7)	−0.0043 (5)
N1	0.0290 (5)	0.0203 (5)	0.0297 (6)	−0.0068 (4)	−0.0013 (4)	−0.0029 (4)
N2	0.0466 (7)	0.0213 (5)	0.0367 (7)	−0.0077 (5)	−0.0003 (5)	−0.0024 (5)
N3	0.0471 (7)	0.0252 (6)	0.0382 (7)	0.0033 (5)	−0.0064 (6)	−0.0005 (5)
C1	0.0325 (6)	0.0206 (5)	0.0275 (6)	−0.0065 (5)	−0.0063 (5)	−0.0023 (5)
C2	0.0303 (6)	0.0206 (5)	0.0255 (6)	−0.0049 (5)	−0.0032 (5)	−0.0017 (5)
C3	0.0349 (7)	0.0297 (6)	0.0254 (6)	−0.0046 (5)	0.0014 (5)	−0.0003 (5)
C4	0.0359 (7)	0.0300 (7)	0.0316 (7)	0.0012 (5)	0.0029 (5)	−0.0050 (5)
C5	0.0329 (7)	0.0224 (6)	0.0320 (7)	−0.0036 (5)	−0.0062 (5)	−0.0025 (5)
C6	0.0497 (9)	0.0280 (7)	0.0263 (7)	−0.0015 (6)	0.0031 (6)	0.0030 (5)
C7	0.0440 (8)	0.0270 (6)	0.0273 (7)	0.0007 (6)	0.0048 (6)	−0.0023 (5)
C8	0.0595 (11)	0.0315 (8)	0.0609 (11)	0.0102 (7)	−0.0030 (9)	−0.0085 (7)
C9	0.0647 (11)	0.0267 (7)	0.0436 (9)	−0.0036 (7)	−0.0102 (8)	0.0054 (6)
C10	0.0279 (6)	0.0217 (5)	0.0267 (6)	−0.0062 (5)	0.0004 (5)	−0.0050 (5)
C11	0.0320 (6)	0.0212 (5)	0.0254 (6)	−0.0085 (5)	0.0062 (5)	−0.0076 (5)
C12	0.0322 (7)	0.0300 (6)	0.0423 (8)	−0.0145 (5)	0.0037 (6)	−0.0110 (6)
C13	0.0299 (7)	0.0349 (7)	0.0494 (9)	−0.0081 (6)	−0.0076 (6)	−0.0067 (6)
C14	0.0325 (7)	0.0240 (6)	0.0409 (8)	−0.0053 (5)	−0.0053 (6)	−0.0016 (5)
C15	0.0373 (7)	0.0209 (6)	0.0304 (7)	−0.0086 (5)	0.0107 (5)	−0.0078 (5)

### Geometric parameters (Å, °)

**Table:**

Zn1—O1	2.0442 (9)	C3—C4	1.3795 (19)
Zn1—O1^i^	2.0442 (9)	C3—H3	0.9300
Zn1—O4	2.1503 (11)	C4—H4	0.9300
Zn1—O4^i^	2.1503 (11)	C5—C4	1.411 (2)
Zn1—N1	2.1963 (10)	C5—C6	1.403 (2)
Zn1—N1^i^	2.1963 (10)	C6—H6	0.9300
O1—C1	1.2691 (16)	C7—C6	1.3802 (19)
O2—C1	1.2624 (17)	C7—H7	0.9300
O3—C15	1.2327 (17)	C8—H8A	0.9600
O4—H41	0.78 (2)	C8—H8B	0.9600
O4—H42	0.81 (2)	C8—H8C	0.9600
O5—H51	0.85 (3)	C9—H9A	0.9600
O5—H52	0.81 (3)	C9—H9B	0.9600
N1—C10	1.3402 (15)	C9—H9C	0.9600
N1—C14	1.3371 (17)	C10—C11	1.3900 (17)
N2—C15	1.3279 (19)	C10—H10	0.9300
N2—H21	0.845 (19)	C11—C12	1.3871 (19)
N2—H22	0.829 (19)	C12—C13	1.382 (2)
N3—C5	1.3675 (17)	C12—H12	0.9300
N3—C8	1.440 (2)	C13—H13	0.9300
N3—C9	1.444 (2)	C14—C13	1.3816 (19)
C1—C2	1.4881 (17)	C14—H14	0.9300
C2—C3	1.3911 (19)	C15—C11	1.5045 (17)
C2—C7	1.3923 (19)		
			
O1^i^—Zn1—O1	180.00 (9)	C5—C4—H4	119.7
O1—Zn1—O4	88.47 (4)	N3—C5—C4	121.30 (13)
O1^i^—Zn1—O4	91.53 (4)	N3—C5—C6	121.57 (13)
O1—Zn1—O4^i^	91.53 (4)	C6—C5—C4	117.13 (12)
O1^i^—Zn1—O4^i^	88.47 (4)	C5—C6—H6	119.4
O1—Zn1—N1	90.96 (4)	C7—C6—C5	121.30 (13)
O1^i^—Zn1—N1	89.04 (4)	C7—C6—H6	119.4
O1—Zn1—N1^i^	89.04 (4)	C2—C7—H7	119.2
O1^i^—Zn1—N1^i^	90.96 (4)	C6—C7—C2	121.51 (14)
O4—Zn1—O4^i^	180.00 (8)	C6—C7—H7	119.2
O4—Zn1—N1	87.36 (4)	N3—C8—H8A	109.5
O4^i^—Zn1—N1	92.64 (4)	N3—C8—H8B	109.5
O4—Zn1—N1^i^	92.64 (4)	N3—C8—H8C	109.5
O4^i^—Zn1—N1^i^	87.36 (4)	H8A—C8—H8B	109.5
N1^i^—Zn1—N1	180.0	H8A—C8—H8C	109.5
C1—O1—Zn1	130.87 (9)	H8B—C8—H8C	109.5
Zn1—O4—H41	102.7 (16)	N3—C9—H9A	109.5
Zn1—O4—H42	117.8 (15)	N3—C9—H9B	109.5
H42—O4—H41	113 (2)	N3—C9—H9C	109.5
H52—O5—H51	106 (2)	H9A—C9—H9B	109.5
C10—N1—Zn1	122.97 (8)	H9A—C9—H9C	109.5
C14—N1—Zn1	119.13 (8)	H9B—C9—H9C	109.5
C14—N1—C10	117.87 (11)	N1—C10—C11	123.24 (12)
C15—N2—H21	120.5 (12)	N1—C10—H10	118.4
C15—N2—H22	123.3 (13)	C11—C10—H10	118.4
H21—N2—H22	115.9 (17)	C10—C11—C15	123.17 (12)
C5—N3—C8	120.47 (14)	C12—C11—C10	117.79 (11)
C5—N3—C9	120.68 (14)	C12—C11—C15	119.02 (11)
C8—N3—C9	118.82 (13)	C11—C12—H12	120.3
O1—C1—C2	116.26 (12)	C13—C12—C11	119.49 (12)
O2—C1—O1	123.66 (11)	C13—C12—H12	120.3
O2—C1—C2	120.08 (12)	C12—C13—H13	120.7
C3—C2—C1	120.65 (12)	C14—C13—C12	118.62 (13)
C3—C2—C7	117.35 (12)	C14—C13—H13	120.7
C7—C2—C1	122.00 (12)	N1—C14—C13	122.98 (12)
C2—C3—H3	118.9	N1—C14—H14	118.5
C4—C3—C2	122.10 (13)	C13—C14—H14	118.5
C4—C3—H3	118.9	O3—C15—N2	122.89 (13)
C3—C4—C5	120.56 (13)	O3—C15—C11	119.01 (13)
C3—C4—H4	119.7	N2—C15—C11	118.10 (12)
			
O4—Zn1—O1—C1	−158.28 (11)	O1—C1—C2—C7	−176.86 (13)
O4^i^—Zn1—O1—C1	21.72 (11)	O2—C1—C2—C3	−177.34 (13)
N1—Zn1—O1—C1	114.39 (11)	O2—C1—C2—C7	2.4 (2)
N1^i^—Zn1—O1—C1	−65.61 (11)	C1—C2—C3—C4	178.22 (13)
O1—Zn1—N1—C14	19.82 (11)	C7—C2—C3—C4	−1.5 (2)
O1^i^—Zn1—N1—C14	−160.18 (11)	C1—C2—C7—C6	−178.48 (14)
O1—Zn1—N1—C10	−162.18 (11)	C3—C2—C7—C6	1.3 (2)
O1^i^—Zn1—N1—C10	17.82 (11)	C2—C3—C4—C5	−0.1 (2)
O4—Zn1—N1—C10	109.39 (11)	N3—C5—C4—C3	−177.74 (14)
O4^i^—Zn1—N1—C10	−70.61 (11)	C6—C5—C4—C3	1.9 (2)
O4—Zn1—N1—C14	−68.60 (11)	N3—C5—C6—C7	177.47 (15)
O4^i^—Zn1—N1—C14	111.40 (11)	C4—C5—C6—C7	−2.2 (2)
Zn1—O1—C1—O2	−33.61 (19)	C2—C7—C6—C5	0.6 (2)
Zn1—O1—C1—C2	145.62 (9)	N1—C10—C11—C12	−0.6 (2)
Zn1—N1—C10—C11	−178.36 (9)	N1—C10—C11—C15	−179.02 (12)
C14—N1—C10—C11	−0.3 (2)	C10—C11—C12—C13	1.3 (2)
Zn1—N1—C14—C13	178.79 (12)	C15—C11—C12—C13	179.73 (14)
C10—N1—C14—C13	0.7 (2)	C11—C12—C13—C14	−1.0 (2)
C8—N3—C5—C4	−1.0 (2)	N1—C14—C13—C12	0.0 (3)
C8—N3—C5—C6	179.36 (15)	O3—C15—C11—C10	165.58 (13)
C9—N3—C5—C4	−179.22 (15)	O3—C15—C11—C12	−12.8 (2)
C9—N3—C5—C6	1.1 (2)	N2—C15—C11—C10	−13.6 (2)
O1—C1—C2—C3	3.40 (18)	N2—C15—C11—C12	168.04 (13)

Symmetry codes: (i) −*x*, −*y*, −*z*.

### Hydrogen-bond geometry (Å, °)

**Table:**

Cg1 is the centroid of the C2–C7 ring.

**Table:**

*D*—H···*A*	*D*—H	H···*A*	*D*···*A*	*D*—H···*A*
N2—H21···O5^ii^	0.85 (2)	2.05 (2)	2.8826 (19)	169.3 (2)
O4—H41···O2^iii^	0.78 (2)	2.00 (2)	2.7370 (16)	159 (2)
O4—H42···O3^iv^	0.81 (2)	1.96 (2)	2.7681 (15)	175.1 (2)
O5—H51···O2	0.85 (3)	2.02 (3)	2.8732 (19)	174 (3)
O5—H52···O2^v^	0.81 (3)	2.11 (3)	2.9150 (18)	173 (2)
C13—H13···O4^vi^	0.93	2.52	3.4422 (19)	170
N2—H22···Cg1^iii^	0.829 (19)	2.79 (2)	3.5200 (15)	147.9 (2)

Symmetry codes: (ii) *x*, *y*+1, *z*−1; (iii) −*x*, −*y*, −*z*; (iv) *x*, *y*−1, *z*; (v) −*x*, −*y*, −*z*+1; (vi) −*x*+1, −*y*, −*z*.
